# Corticosteroids to prevent renal scarring in children with pyelonephritis: a systematic review and meta-analysis

**DOI:** 10.1007/s40620-022-01552-1

**Published:** 2023-01-24

**Authors:** Johanna Jääskeläinen, Marjo Renko, Ilari Kuitunen

**Affiliations:** 1 Institute of Clinical Medicine and Department of Pediatrics, University of Eastern Finland, Yliopistonranta 2, 70211 Kuopio, Finland; 2 Department of Pediatrics, Kuopio University Hospital, Kuopio, Finland; 3 Department of Pediatrics, Mikkeli Central Hospital, Mikkeli, Finland

**Keywords:** Pyelonephritis, Corticosteroids, Renal scars

## Abstract

**Background:**

Acute pyelonephritis is a common infection in children that may cause renal scarring. The aim of this systematic review and meta-analysis was to analyse the use of corticosteroid treatment to prevent renal scarring.

**Methods:**

We searched the PubMED, SCOPUS, Cochrane CENTRAL and Web of Science databases in June 2022 for (corticosteroid* or dexamethasone or prednisolone* or prednisone* or hydrocortisone*) AND pyelonephritis. Randomised controlled trials focusing on children were included. The intervention was corticosteroid treatment with antibiotics compared to antibiotics with or without a placebo. The main outcome was the presence of renal scars on dimercaptosuccinic acid scanning at follow-up. The evidence quality was assessed using the GRADE methodology and risk of bias 2.0 tool. We calculated the risk ratio (RR), absolute risk difference (RD) with 95% confidence intervals (CI) and the number needed to treat (NNT). We applied a fixed effects model due to low heterogeneity.

**Results:**

We screened 872 abstracts and included five full texts. Renal scarring at follow-up was found in 31/220 (14.1%) patients in the corticosteroid groups and 76/278 (27.3%) in the control groups (RR 0.65, CI 0.44–0.96, RD − 13.2%, NNT 8). The evidence quality was moderate. Two studies reported adverse events with no differences between the groups. The risk of bias analysis showed some concerns in four studies.

**Conclusion:**

We found moderate quality evidence that adjuvant corticosteroid treatment could prevent renal scarring. Adverse events were insufficiently reported, and more research on their effectiveness and harm is therefore needed before using corticosteroids in clinical settings.

## Introduction

Urinary tract infections (UTIs) are one of the most common bacterial infections in childhood as approximately 4% of 1-year-olds and 10% of 6-year-olds have an episode of UTI [1]. Acute pyelonephritis may cause renal scarring, and renal scarring has been detected by dimercaptosuccinic acid (DMSA) scanning in approximately 15–57% of patients after the acute phase [2, 3]. Renal scarring may cause later renal sequalae, such as hypertension and kidney insufficiency; however, the relationship is not completely straightforward [4, 5]. Some countries routinely use DMSA scanning, and some countries do not use it at all.

Researchers have sought potential treatments to prevent scarring, and strategies such as corticosteroid therapy, vitamin supplementation and antibiotic treatment have been assessed. Antibiotic treatment alone does not offer sufficient protection from scarring as renal scars seem to develop regardless of the appropriately started antibiotic treatment [3, 6]. The formation of scars seems to originate from the inflammatory process in the kidneys rather than the bacterial infection, thus corticosteroids could potentially offer protection from renal scarring. In recent randomised studies, corticosteroid treatment has produced controversial results with respect to renal scarring. A meta-analysis published in 2021 showed the effects against scarring when corticosteroid therapy was combined with routine antibiotic treatment [7]. Since then, novel studies have been published, and we therefore decided to update the evidence.

The aim of this systematic review and meta-analysis was to analyse whether adjuvant corticosteroid treatment can prevent renal scarring in acute pyelonephritis in children.

## Methods

### Search strategy

We searched the PubMed (MEDLINE), Cochrane CENTRAL, Scopus and Web of Science databases in June 2022. The full search strategy is described in Supplement 1. We included only studies published in English. We did not use any time or other filters. The search results were then uploaded for screening in Covidence software (Covidence, Melbourne, Australia) (Table 1).

**Table 1:** Background information of included studies

Study	Country	Design	Blinding	Participants (*N*)	Intervention	Control	Main outcome	Secondary outcomes
Da Dalt et al. 2021 [15]	Italy	RCT	None	18	Dexamethasone 0.15 mg/kg per dose every 12 h for 4 days and oral amoxicillin-clavulanate for 10 days	Oral amoxicillin-clavulanate for 10 days	Presence of kidney scars on the DMSA scan performed at the 6-month follow up	Kidney scarring in children with higher procalcitonin values, the acceptability of adjuvant steroid treatment (treatment discontinuation, reported side effects)
Ghaffari et al. 2019 [13]	Iran	RCT	Double-blinded	52	Ceftriaxone 80 mg/kg/d and dexamethasone 0.15 mg/kg every 6 h for 4 days intravenously	Ceftriaxone 80 mg/kg/d and placebo (normal saline)	NA	NA
Huang et al. 2011 [12]	Taiwan	RCT	Double-blinded	83	Cephalothin (100 mg/kg per day intravenously) every 6 h and gentamicin (5 mg/kg per day intravenously) every 12 h for a minimum of 3 days and oral methylprednisolone (1.6 mg/kg per day, maximum of 48 mg/day) in divided doses every 6 h	Cephalothin (100 mg/kg per day intravenously) every 6 h and gentamicin (5 mg/kg per day intravenously) every 12 h for a minimum of 3 days. When patients were discharged after being afebrile for 2 days, parenteral antibiotics were changed to oral antibiotics for approximately 14 days. Low dose cephalothin or trimethoprim was given for 2–4 weeks until VGUG was performed	Development of renal scars in follow-up DMSA scan 6 months after enrolment	NA
Rius-Gordillo et al. 2021 [16]	Spain	RCT	Double-blinded	91	Intravenous dexamethasone 0.30 mg/l twice a day for 3 days in addition to empirical parenteral antibiotic treatment forts, then oral antibiotic treatment	Placebo and empirical parenteral antibiotic treatment, then oral antibiotic treatment	Kidney scarring in DMSA scan 6 months after enrolment	Kidney scarring risk factors (vesicoureteral reflux, congenital anomalies of the kidney, or urinary tract dilatation)
Shaikh et al. 2020 [14]	USA	RCT	Double-blinded	254	Oral dexamethasone (0.15 mg/kg) twice daily for 3 days and 10 days of antibiotics chosen by the treating physician for urinary tract infection	Placebo and 10 days of antibiotics chosen by the treating physician for urinary tract infection	Kidney scarring and severe kidney scarring in DMSA scan performed 5–24 months after enrolment	NA

### Inclusion and exclusion criteria

We included randomised controlled trials (RCTs) that compared adjuvant corticosteroid treatment to standard antibiotic treatment with or without a placebo, as well as studies conducted on children aged older than 28 days but younger than 18 years. Animal studies were excluded as were observational studies and all other studies that did not present original data (reviews, editorials, letters, etc.).

### Review process

**Fig. 1:**
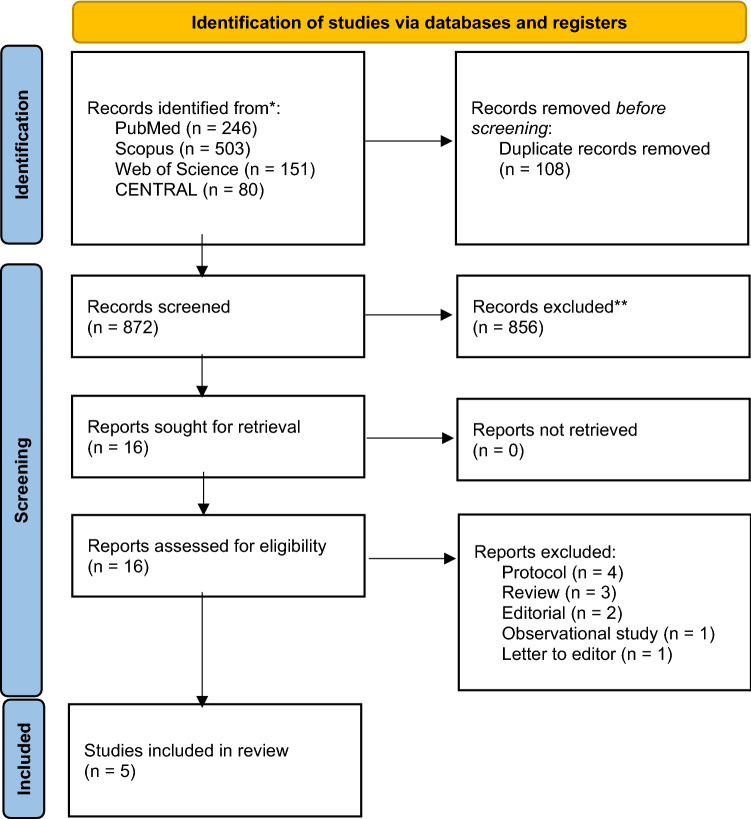
Flow chart of the study selection process

Two authors (JJ and IK) individually screened the abstracts and conflicts were resolved by the third author (MR). Full texts were assessed independently by two authors (IK and JJ). The outcomes data was then extracted into an Excel spreadsheet. The Cochrane risk of bias 2.0 tool was used to assess risk of bias in the included studies. The risk of bias figures were created by using the robvis package in R version 4.0.3. The evidence quality was assessed using the Grading of Recommendations Assessment, Development and Evaluation (GRADE) methodology [8] (Fig. 1).

### Outcomes measures

Our primary outcome was the presence of renal scarring on DMSA scans after the acute phase. Our secondary outcomes were the severity of the scarring on DMSA scans and adverse events related to the initial corticosteroid treatment (Table 2).

**Table 2:** Inclusion and exclusion criteria in the included studies

Study	Inclusion criteria	Definition of UTI	Exclusion criteria
Da Dalt et al. 2021 [15]	Children, aged 2–24 months with first episode of febrile urinary tract infection, PCT levels above 1 ng/ml indicating high risk for kidney scarring	Axillary temperature > 37.5 °C in children with no other signs of infection and positive dipstick on urine samples collected by urine catheterization	Antibiotic treatment in the 48 h before evaluation, diagnosed underlying kidney diseases or urinary tract abnormalities, a previous urinary tract infection, urinary tract infection recurrence before second DMSA scan, prematurity, immunodeficiency, contraindication to steroid therapy, negative urine culture
Ghaffari et al. 2019 [13]	Febrile children diagnosed with acute pyelonephritis	Fever, with or without urinary tract symptoms, an in urinary analysis leukocyte esterase, white blood cells, bacteria and nitrite, positive urine culture, urine samples were obtained by suprapubic bladder aspiration, or obtained from midstream clean-catch specimen or catheter specimen	Previous urinary tract infection, urinary tract abnormalities, taking antibiotics, renal scars, renal failure,
Huang et al. 2011 [12]	Children aged 1 week to 16 years, with evidence of UTI, were at high risk of renal scar formation, DMSA performed within 48–72 h after admission	Fever (38 °C) and positive urine culture, defined as 5 leukocyte cells per high-power field, 105 colony-forming units/ml in midstream clean-catch specimen and 103 colony-forming units/ml in catheter specimen or in urine sample taken by suprapubic bladder aspiration	Previous urinary tract infections, previous treatment with antibiotics, known urogenital uropathy (excluding vesicoureteral reflux), DMSA not performed within 72 h of admission, negative findings in DMSA or ultrasonography
Rius-Gordillo et al. 2021 [16]	Children aged 1 month to 14 years with first episode of febrile urinary tract infection	Positive urine culture, urine sample taken by catheterization in non-continent children (> 10,000 CFU/mL), clean catch specimen in continent children (100,000 CFU/mL)	Endocrinological disease, immunosuppression treatment, cancer, or known uropathy, patients with normal acute DMSA, reoccurrence of urinary tract infection before the second DMSA
Shaikh et al. 2020 [14]	Children aged 2 months to 6 years with first episode of febrile urinary tract infection, fever had to be documented	Pyuria in a urinalysis (≥ 5 WBC/hpf in a centrifuged specimen, ≥ 10 WBC/mm3 in an uncentrifuged specimen, or ≥ 1 + leukocyte esterase on dipstick)	Not first UTI, antibiotics in past 7 days, chronic disease, genitourinary anomaly, bagged urine, pneumonia, meningitis or sepsis, steroids in the past 14 days, immune deficiency, Kawasaki disease, allergy to dexamethasone

### Statistics

We used Review Manager version 5.4 for the meta-analysis. The data analyses were performed according to the Cochrane Handbook of Systematic Review Guidelines [9]. We calculated the risk ratios (RR) with 95% confidence intervals (CI) for the dichotomous outcomes and the absolute risk difference (RD) and number needed to treat (NNT) for the primary outcome. A forest plot was presented for the primary outcome and a funnel plot was used to analyse possible publication bias. As the secondary outcomes were measured differently, we decided not to pool them to a single estimate. Instead, they are presented in accordance with the Synthesis Without Meta-analysis (SWiM) guidelines [10]. We analysed the inconsistency index (*I*^2^) statistics for heterogeneity, and as heterogeneity was low (less than 40%), we used a fixed effects model.

We reported our systematic review and meta-analysis according to the Preferred Reporting Items for Systematic Reviews and Meta-Analyses (PRISMA) guidelines (Supplement 2) [11].

### Protocol registration

We registered our protocol in Prospero (CRD42022339721).

## Results

We initially screened 872 abstracts and assessed 16 full texts, with 5 studies selected for the final analysis. These 5 studies included a total of 498 children, with a mean age ranging from 7.4 to 34.19 months [12–16]. The majority of patients were girls, and the most common causative bacteria was *Escherichia coli* (Table 3). A total of 227 children underwent baseline DMSA scan. Da Dalt et al. performed a follow-up DMSA at 6 months after diagnosis, Ghaffari et al. between 4 and 6 months, Huang et al. between 6 and 38 months, Rius-Gordillo et al. at 6 months and Shaikh et al. between 5 and 24 months after diagnosis. Altogether, 182 children were lost to follow up; 130 children in Shaik et al., 21 children in Rius-Gordillo et al., 22 children in DaDalt et al. and 1 child in Huang et al.. Graffari et al. did not specify why 8 children dropped out of the study. Da Dalt, Ghaffari, Rius-Gordillo and Shaikh et al. used dexamethasone as intervention in their RCTs, and doses were 0.15 mg/kg every 12 h for 4 days, 0.15 mg/kg every 6 h for 4 days, 0.30 mg/kg every 12 h for 3 days and 0.15 mg/kg every 12 h for 3 days, respectively. Huang et al. used methylprednisolone 1.6 mg/kg/day with maximum dose of 48 mg/day in divided doses every 6 h for 3 days.

**Table 3:** Study population baseline characteristics in the included studies

Study	*N* of participants (study/control)	Patient age months (mean)		Gender male		*E. coli*	
		Study	Control	Study	Control	Study	Control
Da Dalt et al. 2021 [15]	18 (7/11)	9.4 (5.3–12.3)	7.4 (3.7–13.7)	4	4	20 (87%)	22 (88%)
Ghaffari et al. 2019 [13]	52 (23/29)	34.19 ± 30.82	50.55 ± 44.41	2	2	NA	NA
Huang et al. 2011 [12]	83 (18/65)	87	8	10	34	17 (89.5%)	55 (84.6%)
Rius-Gordillo et al. 2021 [16]	91 (49/42)	8.0	9.5	12	12	42 (100%)	47 (96%)
Shaikh et al. 2020 [14]	254 (123/131)	17.2	20.8	14	17	117 (95%)	121 (92%)

### Risk of bias

**Fig. 2:**
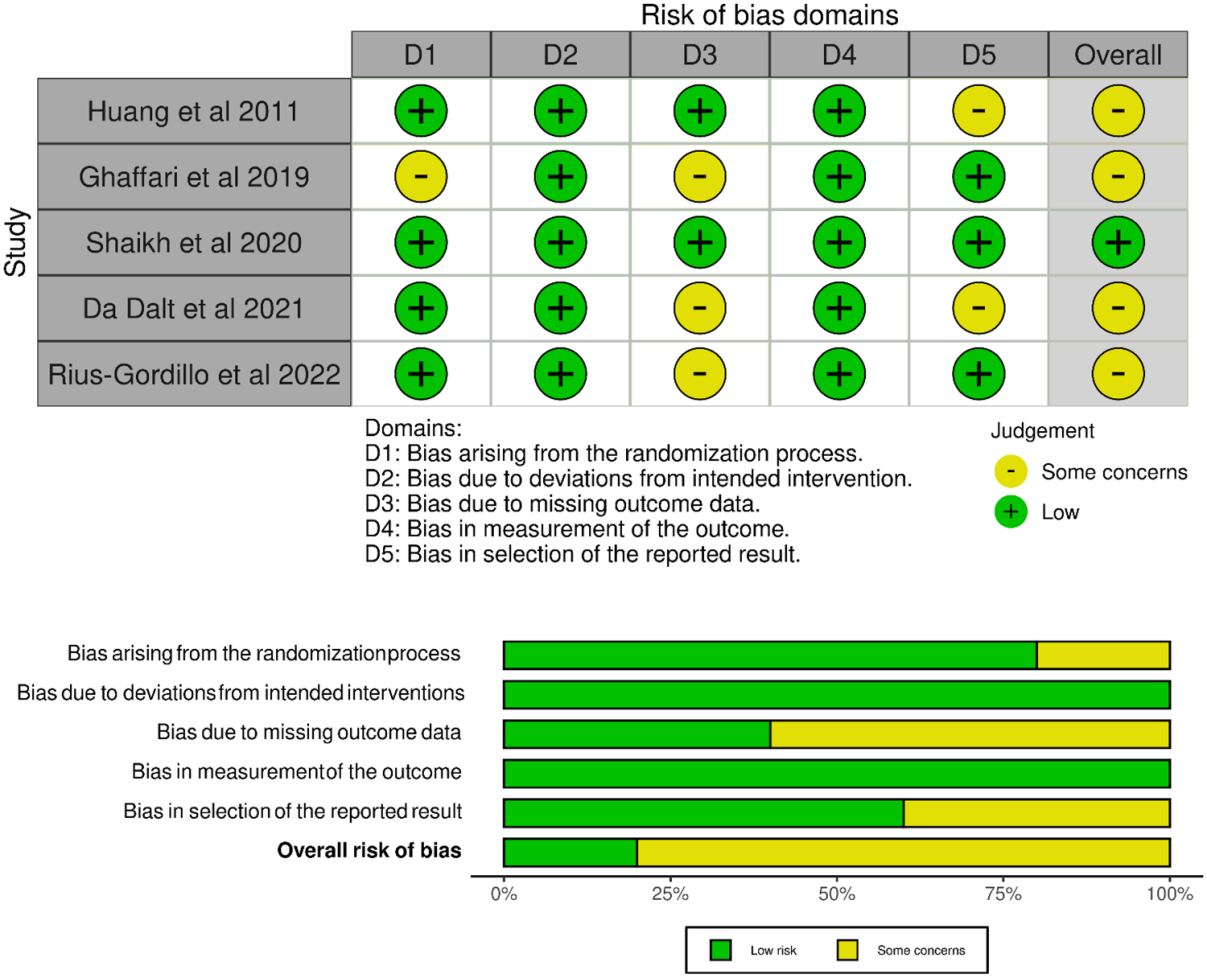
Risk of bias in the included studies assessed in five domains and overall

The overall risk of bias was considered low in one study although there were some concerns regarding bias in four studies (Fig. 2A). Most of the bias was due to missing outcomes data and the selection of the reported results (Fig. 2B).

### Renal scarring

**Fig. 3:**
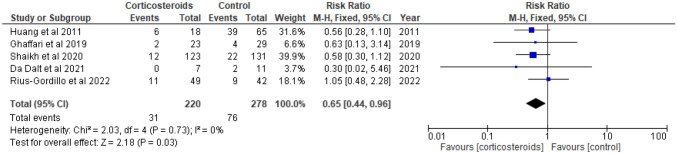
Forest plot

**Fig. 4:**
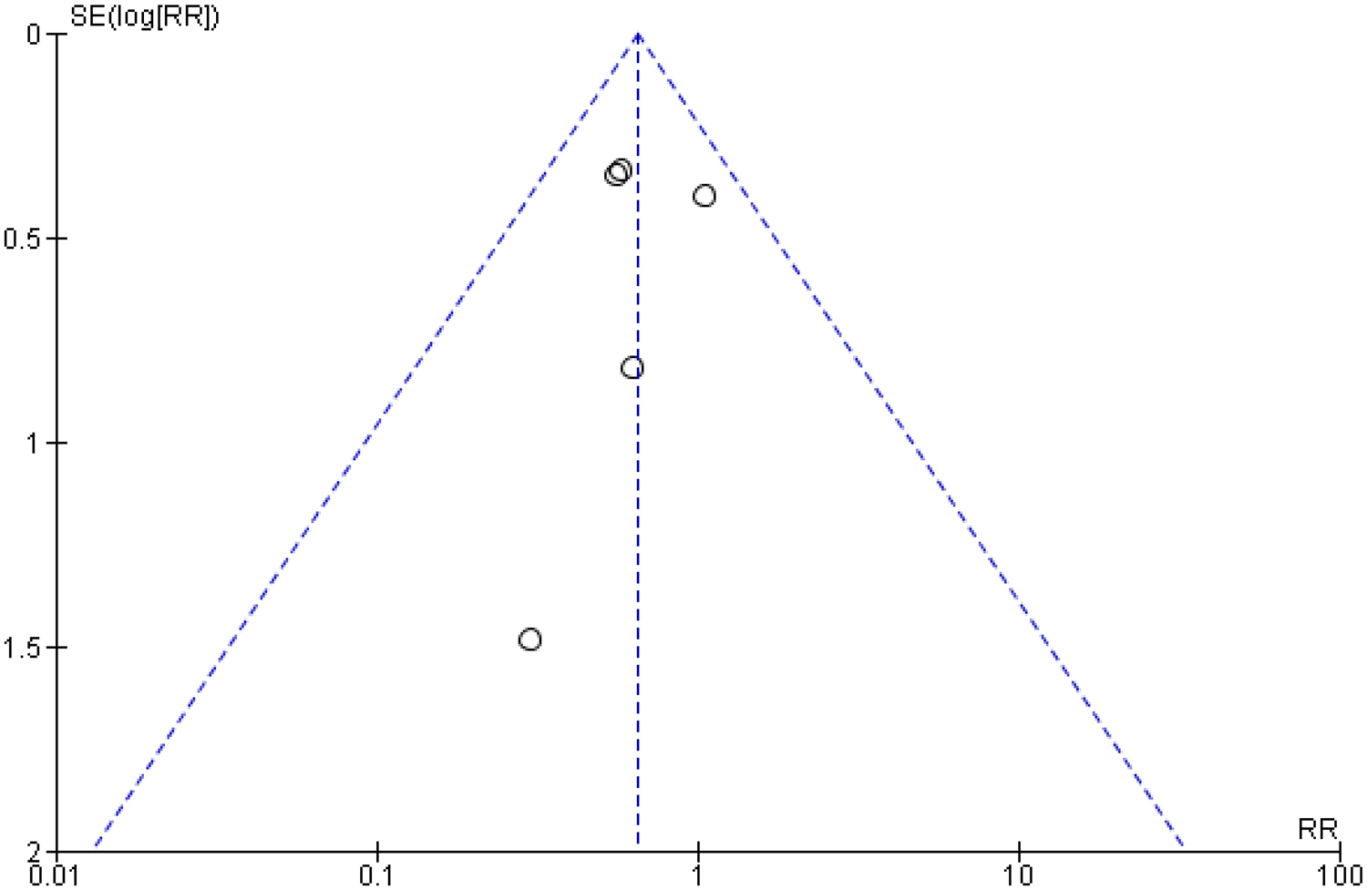
Funnel plot

Renal scarring was found in 31 (14.1%) of the 220 children in the corticosteroid groups and 76 (27.3%) of the 278 children in the control groups (RR 0.65 [CI 0.44–0.96], absolute RD − 13.2%, NNT 8; Fig. 3, Table 4). We ranked the evidence quality as moderate due to the risk of bias (Table 4). The funnel plot was symmetrical, and publication bias was not observed (Fig. 4).

**Table 4:** Body of evidence for outcomes assessed by the GRADE methodology and summary of the findings table

Outcome	Quality assessment							Summary of findings				
								*N* of patients		Effect		Quality of evidence
	*N* of studies	Design	Risk of bias	Inconsistency	Indirectness	Imprecision	Publication bias	Intervention *n*/*N*	Control *n*/*N*	Relative risk (95% CI)	Absolute risk difference (95% CI)	
Renal scarring	5	RCT	Some concerns due to missing outcome data and selection of reporting	No serious limitations	Not present	No serious limitations	Not present	31/220	76/278	0.65 (0.44–0.96)	− 13.2% (−20.2 to −6.3%)	Moderate
Synthesis without meta-analysis												
renal scarring severity^1^	3	RCT	Some concerns due to missing outcome data and selection of reporting	No serious limitations	Not present	No serious limitations	Not present	The median volume of renal scarring was 0.0 ml and was found in fewer segments, there was no significant difference in kidney scarring severity score between the groups	The median volume of renal scarring was 1.5 ml and renal scarring was found in 2.4 segments	–	–	Low
Adverse events^2^	2	RCT	Some concerns due to missing outcome data and selection of reporting	No serious limitations	Not present	No serious limitations	Not present	Fussiness 9.2%, serious adverse events 2.6%, one case of transient behavioural change	Fussiness 2.9%, serious adverse events 2.9%	–	–	Low

^1^Studies were judged to be unsuitable for meta-analysis due to heterogeneous outcome measurement

^2^Studies were judged to be unsuitable for meta-analysis due to heterogeneous outcome measurement

### Renal scarring severity

Renal scarring severity was reported in three studies with 428 children [12, 14, 16]. Analysis of renal scarring volume in the study by Huang et al. found that the median volume was 0.0 ml (range 0.0–4.5 ml) in the corticosteroid group and 1.5 ml (range 0–14.8 ml) in the control group (*p* < 0.01) [12]. In the Shaikh et al. study, kidney scarring was present in 1.9 segments in the corticosteroid group and in 2.4 segments in the control group (*p* = 0.22). Rius-Gordillo et al. analysed renal damage severity scores (range 0–8) in their study. In the follow-up DMSA scans, the score was 0.41 in the corticosteroid group and 0.32 in the control group (*p* = 0.848) [14, 16].

### Adverse events

Two studies comprising a total of 272 children reported adverse events [14, 15]. In the Shaikh et al. study, the non-severe and severe adverse outcome rates were similar between the corticosteroid and control groups, and the serious adverse events (i.e., hospitalisations and bacteraemia findings) were similar in both groups (2.6% vs. 2.9%, respectively) [14]. Da Dalt et al. reported that there was only one case of transient behavioural change in the corticosteroid group in their study, and no other adverse outcomes were observed [15].

## Discussion

This meta-analysis consisted of five studies that included 498 children, 220 of whom received adjuvant corticosteroid treatment. Renal scarring was found in 14.1 and 27.3% of the patients in the corticosteroid and control groups, respectively. Dexamethasone was the most used steroid in the RCTs included and it seemed to reduce the risk of renal scarring. Methylprednisolone was used in Huang et al. and they found that it reduced renal scaring in the study group [12]. The route of administration of steroids does not seem to affect the risk reduction as both oral and intravenous corticosteroids reduced renal scar formation. When possible, oral administration of corticosteroids should be primary as it is non-invasive. Meena et al. [7] found a reduction in kidney scarring in their fixed-effects meta-analysis (RR 0.57, CI 0.36–0.91), which was similar to the findings of our meta-analysis (RR 0.65, CI 0.44–0.96) [7]. The Meena et al. meta-analysis included three RCTs with 389 children, and those three RCTs were also included in our meta-analysis [12–14]. Although it seems that corticosteroids reduced kidney scarring, the reporting of adverse events was poor. Only two studies [14, 15] reported adverse events: fussiness was more common in the corticosteroid group, and one case of transient behavioural change was reported in the corticosteroid group in one study. Although we found no significant differences in the serious adverse events between the corticosteroid and control groups (2.6% vs 2.9%, respectively) in our meta-analysis, these were only reported in one study [14]. Follow-up DMSA scans were done approximately 6 months after recruitment. Long follow-up periods may lead to increased number of dropouts and reinfections. Shaikh et al. had the highest number of patients lost to follow up, and outcome assessment was done 5–24 months after diagnosis [14]. At the 6-month follow up, the rate of dropouts and reinfections remained moderate.

The follow-up studies suggest that renal scarring may lead to the development of hypertension, renal insufficiency and an increased risk of preeclampsia during pregnancy [4, 5, 17]. Jacobson et al. found a significant reduction in the glomerular filtration rate (GFR) and higher diastolic blood pressure in the patients with kidney scars than in the control patients [4]. However, in more recent studies such findings have not been as clear [18]. Wennerström et al. did not find any significant difference in ambulatory blood pressure between the children with non-obstructive renal scarring after the first UTI and the reference group without renal scarring [5]. Notwithstanding, there was an increase in atrial natriuretic peptin in the study group, which suggests that patients with renal scars could have an ongoing counter-regulatory mechanism that prevents blood pressure from increasing. After two decades, the GFR was well preserved, but when looking at the function of a single kidney, the GFR was significantly lower in the scarred kidney than in the healthy one [17]. Evidence suggests that children with kidney scars are at a higher risk of developing hypertension and kidney insufficiency later in life.

We were able to conduct our review in line with our protocol, which is a strength of our study. The limitations of this meta-analysis arose mostly from the original publications. The main limitation of the included studies was the limited number of participants and high dropout rates. First, one of the studies changed the prespecified analysis plan to the Bayesian approach during the study process [15]. Second, most of the studies did not discuss potential adverse events or harm stemming from corticosteroid treatment. Only one of the studies included confirmation of pyelonephritis via DMSA scans, and in one study, pyelonephritis was confirmed via either DMSA scans or renal sonography [12, 16]. Although the incidence of pyelonephritis from febrile UTIs has been shown to be approximately 70%, in the three studies where pyelonephritis was defined as febrile UTIs, one third of the patients included may not have had true acute pyelonephritis [6, 13–15]. This could have affected the final results in those three studies. The lack of baseline DMSA scans makes it impossible to discover if the scars were due to acute pyelonephritis or if the children were born with them.

Based on the findings of this meta-analysis, adjuvant corticosteroid therapy could prevent renal scarring in acute pyelonephritis. However, many issues have to be clarified before recommending the use of corticosteroids in clinical settings. First, the diagnosis of pyelonephritis should be sound before considering steroids. Second, we need more information on the side effects of the steroid treatment with this indication. Furthermore, clinical studies are needed to determine the optimal dose of corticosteroids with ideal risk/benefit ratio. Third, the real clinical impact of the scars should be elucidated with effective and long monitoring. Baseline DMSA scans before the infection episode would probably not be a realistic aim, but an appropriate randomization process is needed to undertake this and other baseline biases. These issues should be taken into consideration in future trials.

## Conclusions

We found moderate quality evidence supporting that adjuvant corticosteroid treatment could prevent renal scarring in acute pyelonephritis in children. The NNT to avoid single renal scarring was eight in our analysis. However, the reporting of adverse events was considered to be insufficient, and more research on the effectiveness and potential harm of corticosteroid treatment is therefore needed before considering routinely using corticosteroids in all clinical settings.

## Data Availability

All the applied materials and analyses are provided in the Supplements or can be requested directly from the corresponding author.
